# Acupuncture‐Related Therapies as a Potential Adjuvant Option for Parkinson’s Disease: Effects on Symptom Management, Medication Use, and Mortality

**DOI:** 10.1155/padi/5512318

**Published:** 2026-06-17

**Authors:** Ye-Seul Lee, Han-Gyul Lee, Hi-Joon Park, Seungwon Kwon, Bo-Hyoung Jang

**Affiliations:** ^1^ Jaseng Spine and Joint Research Institute, Jaseng Medical Foundation, Seoul, South Korea, jaseng.org; ^2^ Department of Cardiology and Neurology, Kyung Hee University College of Korean Medicine, Kyung Hee University Medical Center, Seoul, South Korea, khmc.or.kr; ^3^ Acupuncture and Meridian Science Research Center, College of Korean Medicine, Kyung Hee University, Seoul, South Korea, khu.ac.kr; ^4^ Department of Preventive Medicine, College of Korean Medicine, Kyung Hee University, Seoul, South Korea, khu.ac.kr

**Keywords:** acupuncture, mortality, national database, nonmotor symptoms, Parkinson’s disease

## Abstract

**Background:**

There is increasing demand for an effective adjuvant therapies for Parkinson’s disease (PD) patients to minimize side effects and improve quality of life, due to unmet needs not fully addressed by conventional treatments.

**Objectives:**

To examine the long‐term effects of adjuvant acupuncture‐related therapies on medication use and mortality in patients with PD using the National Health Insurance Service (NHIS) database in Korea.

**Methods:**

NHIS records from 2010 to 2011 were searched to extract the study population. The medical records of patients with PD were followed from the initial diagnosis to August 2018. Propensity score matching was performed using covariates, including age group, sex, and duration of levodopa therapy. Odds ratios (ORs) of levodopa therapy and nonmotor symptom medication use were examined. Cox proportional hazards modeling and Kaplan–Meier analysis were performed to determine differences between acupuncture‐related therapy users (ACU group) and nonusers (non‐ACU group).

**Results:**

The ACU group (*n* = 23,454) used less levodopa therapy compared to non‐ACU group (*n* = 23,454) but showed higher use of nonmotor symptom medications including those for sleep disorders, anxiety, and depression. In the Cox proportional hazards model, the ACU group was associated with a slightly lower long‐term risk of all‐cause mortality.

**Conclusion:**

Acupuncture‐related therapies—defined here as one or more treatments delivered at Korean medicine clinics, including acupuncture, electroacupuncture, moxibustion, cupping, and herbal formulations—were more frequently received by patients with nonmotor symptoms. The use of levodopa therapy and all‐cause mortality was lower in the ACU group than in the non‐ACU group. Given the observational design, heterogeneity of the exposure, and potential residual confounding, these associations should not be interpreted as causal evidence of therapeutic benefit. Prospective, randomized, sham‐controlled trials are required to determine whether acupuncture‐related therapies confer specific clinical benefit in patients with PD.

## 1. Introduction

Parkinson’s disease (PD) is a prevalent neurodegenerative disease among people over the age of 65 [1–3], affecting more than 10 million people worldwide [4]. The major clinical symptoms include motor symptoms, such as bradykinesia, resting tremor, postural instability, and muscular rigidity, and nonmotor symptoms, such as cognitive dysfunction, anxiety, depression, and sleep disorders [1, 3, 5–8]. In Korea, the number of patients with PD has more than doubled from 39,265 in 2004 to 96,499 in 2016 [9], and the hazard ratio (HR) for mortality in the PD patient group was 2.09 compared to non‐PD controls [10].

While the introduction of levodopa led to improvements in the symptom management of PD, the mortality of post‐levodopa patients varied, often with moderate‐to‐severe side effects. Therapies such as physical exercise and additional treatments such as deep brain stimulation have been suggested with limited results. These findings highlight the need for adjuvant treatments that minimize the side effects and improve the survival and quality of life of patients with PD.

In this regard, the effects of traditional, complementary, and integrative medicine (TCIM) therapies—including those rooted in traditional Chinese medicine (TCM), recognized by the WHO, such as acupuncture, moxibustion, cupping, and herbal medicine—as well as other approaches like massage therapy, have been investigated in patients with PD. Despite concerns of moderate‐ to low‐quality evidence [11] and methodologies of existing studies [12, 13], the literature shows that acupuncture‐related therapies as an adjuvant therapy improve various PD symptoms, such as cognitive deficits, motor symptoms [11–17], and patients’ quality of life [18]. The main reason for the use of TCIM therapies in PD patients was reported to be the unmet need to manage symptoms not fully addressed by conventional treatments [19–22], which have led to the recent studies on acupuncture utilization in patients with PD by clinicians and researchers across several countries [20–23].

Studies researching PD in recent years have focused on nonmotor symptoms, which often occur in early stages of the disease and dominate all stages. Nonmotor symptoms have a serious impact on the quality of life [24], and these symptoms include cognitive dysfunction, olfactory deficits, anxiety, depression, and sleep disorders [25], most of which are treated with medications [26, 27]. Exploration of medications prescribed to treat nonmotor symptoms showed initiation of medication use at an early stage of PD, long duration of use [26], and use of multiple medications [28]. Medication to treat cognitive decline has been discussed in terms of its appropriateness [29, 30]. While the need for additional medications in patients with PD for nonmotor symptoms is unanimously accepted, this may also increase the duration of stay [28] and possibly increase reported falls [31]. Taken together, patients receiving medication for nonmotor symptoms may require additional treatment and observation [32].

To further investigate the use of certain therapies by patients with PD and its long‐term effectiveness and possible underlying factors, the national healthcare database is a promising option. In Korea, the National Health Insurance Service (NHIS) is a single insurer covering the whole population with 178 regional branches, and the Big Data Steering Department in the NHIS oversees the provision and analysis of the NHIS database, which was initiated in 2012 using information from the eligibility data, medical treatment records, and health screening data [33]. A previous study reported the mortality and cause of death in patients with PD over a 12‐year period in Korea using a national sample cohort provided by the NHIS [10]. However, the use of acupuncture‐related therapies in Korean patients with PD has not been observed at the national level.

The objective of this study was to examine the long‐term effects of adjuvant acupuncture‐related therapies such as acupuncture and herbal medicine on PD patients’ medication regimen. We examined the characteristics of Korean patients with PD and evaluated the mortality, use of levodopa therapy, and use of nonmotor symptom medications between patients with PD who used acupuncture‐related therapies (ACU group) and those who did not (non‐ACU group). Finally, we explored the possible association between demographic variables and the duration of levodopa therapy with mortality between the ACU and non‐ACU groups.

## 2. Methods

### 2.1. Data Source

NHIS records from 2010 to 2018 were used in this study. The NHIS database is the claims database from the NHIS and represents the entire Korean population [34]. It allows a complete enumeration survey of the Korean population on a real‐world basis [33]. As of December 2017, 50,940,885 people were insured under the NHI and 1,485,740 low‐income Medical Aid beneficiaries [35]. The NHIS database contains nationwide insurance claims data, capturing patient demographics, diagnostic information, treatment histories, medication prescriptions, and healthcare facility identifiers. These records, sourced from both inpatient and outpatient encounters, provide a comprehensive basis for longitudinal health research [36]. Korean Classification of Diseases Seventh Revision (KCD‐7), based on the International Classification of Diseases, 10th Revision, Clinical Modification, was used to code the principal and additional diagnoses. Informed consent was not required for this study, as it utilized retrospective data with encrypted personal identifiers. All data were de‐identified at the point of provision by NHIS, and no individual‐level identifying information was accessible. The Institutional Review Board of Kyung Hee University formally granted a waiver of informed consent. The study was conducted in compliance with the Declaration of Helsinki and applicable Korean legislation on the protection of personal health information.

### 2.2. Study Population

Korean population from 2010 to 2011 was used to define the study population for PD. In 2010, 50,581,191 people were insured under the NHIS and Medical Aid; in 2011, 51,169,141 people were insured under the NHIS and Medical Aid [35]. Inclusion criteria were age above 40 years and the presence of primary parkinsonism or idiopathic PD in the principal or additional diagnosis (KCD‐6 code F023, G20, or G258), according to the main sickness codes or health insurance coverage expansion codes (V124) of NHIS database from 2010 to 2011, and who received antiparkinsonian medication for more than 7 days at the inpatient or outpatient clinic, including levodopa, catechol‐O‐methyltransferase (COMT) inhibitors, dopamine antagonists, monoamine oxidase B (MAO‐B) inhibitors, anticholinergics, and N‐methyl‐D‐aspartate (NMDA) receptor antagonists. The medication inclusion criterion was implemented to enhance the diagnostic validity of PD. The exclusion criteria were death within 1 year of diagnosis, diagnosis of secondary parkinsonism, and a history of any type of parkinsonism or dementia in the past 5 years. The date of the first visit was designated as the index date for data analysis.

Patients in the ACU group were defined as those who had at least one record of receiving treatment at an inpatient or outpatient Korean medicine clinic after the initial diagnosis of PD. In the dual‐medical license system of Korea, doctors of Western and Korean medicines separately diagnose and treat patients, during which consultations and referrals are made if necessary. Since both medical treatments are reimbursed by the NHIS, a cross‐identification of patients could define groups of patients who visited only Western medicine clinics and those who visited both clinics. In this study, patients who visited Korean medicine clinics after the diagnosis of PD and prescription of levodopa at Western medicine clinics were classified as the ACU group, and those who visited only Western medicine clinics were classified as the non‐ACU group. In both groups, patients were diagnosed and treated by state‐licensed clinicians with certified credentials and proven diagnostic expertise.

**FIGURE 1:**
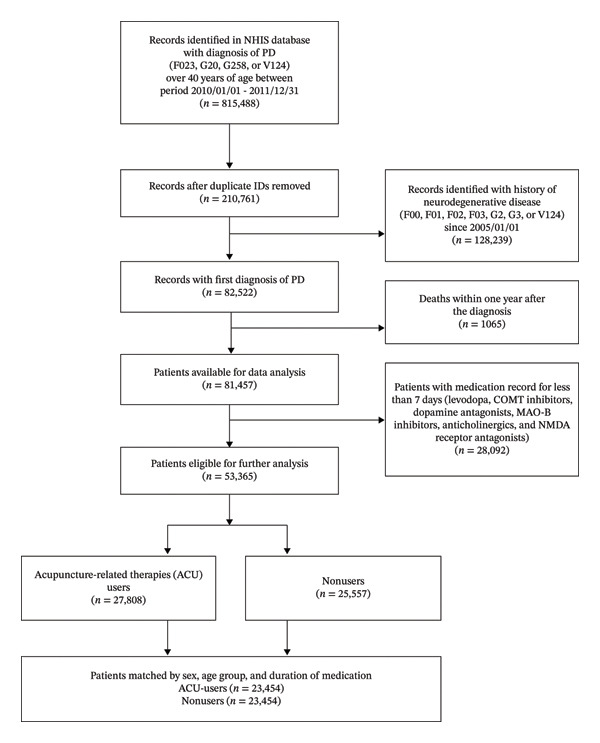
Flowchart of patient selection and matching.

From the aforementioned criteria, patients who were diagnosed with PD in 2010 and 2011 were 53,365 in total and eligible for further analyses. Of these patients, 27,808 (52.1%) received acupuncture‐related therapies, whereas 25,557 (47.9%) did not use any acupuncture‐related treatment until August 2018. Figure 1 illustrates the patient‐selection process from the NHIS database in Korea.

### 2.3. Intervention

The reimbursement records in the NHIS database from the initial diagnosis of PD to August 2018 were examined to include patients with treatment records in Korean medicine clinics in the cohort. Only patients with records of receiving acupuncture‐related therapies after the initial diagnosis of PD were included in the cohort. Acupuncture users underwent one or more traditional therapies—such as acupuncture, electroacupuncture, moxibustion, cupping, or prescribed herbal formulations—administered by certified Korean medicine practitioners, the sole providers authorized to claim NHIS reimbursement for such treatments [37]. Table 1 outlines the types of interventions received by patients with PD in the ACU group. Both the ACU and non‐ACU groups in the PD cohort were followed from the index event until August 2018 to determine all‐cause mortality.

**TABLE 1:** Baseline characteristics of propensity score‐matched Parkinson’s disease cohort with and without ACU treatments.

		Non‐ACU group (*n* = 23,454)		ACU group (*n* = 23,454)		*p* value
		*n*	%	*n*	%	
Year	2010	9020	38.46	13,100	55.85	< 0.001^∗^
	2011	14,434	61.54	10,354	44.15	

Age	40–49	2796	11.92	2761	11.77	0.1161
	50–59	4684	19.97	4777	20.37	
	60–69	6316	26.93	6316	26.93	
	70–79	7163	30.54	7268	30.99	
	80–	2495	10.64	2332	9.94	

Sex	Male	9285	39.59	9283	39.58	0.9925
	Female	14,169	60.41	14,171	60.42	

Region	Gangwon	986	4.20	875	3.73	< 0.001^∗^
	Gyeonggi	4420	18.85	4059	17.31	
	Gyeongnam	1371	5.85	1385	5.91	
	Gyeongbuk	1596	6.80	1839	7.84	
	Gwangju	726	3.10	785	3.35	
	Daegu	1189	5.07	1355	5.78	
	Daejeon	668	2.85	872	3.72	
	Busan	1411	6.02	1594	6.80	
	Seoul	4522	19.28	4181	17.83	
	Ulsan	343	1.46	442	1.88	
	Incheon	1048	4.47	891	3.80	
	Jeonnam	1784	7.61	1674	7.14	
	Jeonbuk	1186	5.06	1228	5.24	
	Chungnam	1445	6.16	1490	6.35	
	Chungbuk	759	3.24	784	3.34	

Duration of levodopa therapy (days)	1	10,156	43.30	10,167	43.35	0.7503
	2	3433	14.64	3369	14.36	
	3	2044	8.71	2027	8.64	
	4	1473	6.28	1439	6.14	
	5	6348	27.07	6452	27.51	

Economic status	Low	5553	23.68	5137	21.90	0.2133
	Moderate	3251	13.86	3046	12.99	
	High	11,701	49.89	12,612	53.77	
	N/A	2949	12.57	2659	11.34	

*Note:* ACU: acupuncture‐related therapies; DTH: death of the patient.

^∗^ *p*‐value < 0.05.

### 2.4. Symptom Severity

To assess the severity of PD in the study cohort, information regarding the types of prescribed medications and duration of levodopa therapy were collected. The prescribed medications included levodopa/carbidopa, levodopa/benserazide, levodopa/carbidopa/entacapone, bromocriptine, ropinirole, and pramipexole (Supporting Table 1). The duration of levodopa therapy since the initial diagnosis was assessed in five categories: (1) 100 days or shorter, (2) 101–200 days, (3) 201–300 days, (4) 301–400 days, and (5) > 400 days.

### 2.5. Outcomes

The outcomes of this study included the all‐cause mortality of patients until August 2018 from the mortality data provided by the NHIS using the Cox proportional hazards ratio and Kaplan–Meier curve analysis. Covariates in the Cox proportional hazards ratio analysis were the use of acupuncture‐related therapies, sex, age group, types of levodopa, and duration of levodopa therapy. The covariate in the Kaplan–Meier curve analysis was the use of acupuncture‐related therapies. The use of medication was compared in the frequency of prescriptions by different forms of levodopa and medications to treat nonmotor symptoms of PD, such as sleeping problems, panic and anxiety disorders, and depression. The odds ratios (ORs) and confidence intervals (CIs) for each prescription of these drugs were calculated.

### 2.6. Statistical Analysis

Chi‐square tests were used to compare the distributions of socioeconomic variables such as age, sex, region of residence, and economic status, represented by health insurance payments between the groups with and without acupuncture‐related treatments. The distribution of medical variables, such as the types of levodopa and duration of levodopa therapy, were tested between the two groups. For the ACU group, the observation period was aligned with the patient’s first clinic visit for acupuncture to minimize the risk of immortal time bias.

**FIGURE 2:**
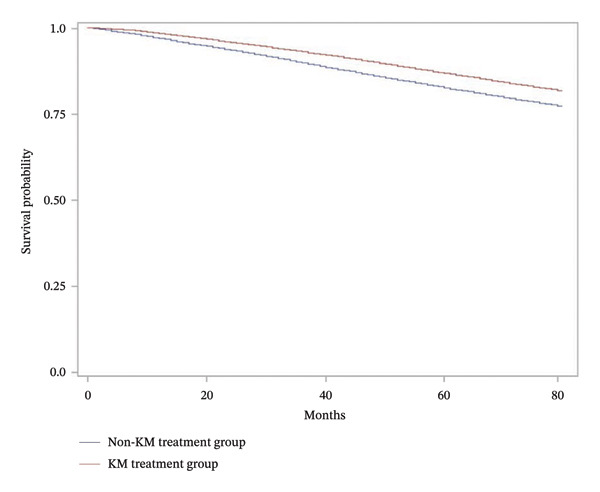
Kaplan–Meier estimates of all‐cause survival in the unmatched Parkinson’s disease cohort with and without adjuvant acupuncture‐related therapies. Note: this Kaplan–Meier curve was generated from the pre‐matching cohort and is presented for descriptive comparison. The primary inference for the survival outcome is based on the propensity score‐matched, covariate‐adjusted Cox proportional hazards model (adjusted HR: 0.90, 95% CI: 0.89–0.91; Supporting Table 3). Log‐rank test, *χ* ^2^ = 1219.45, *p* < 0.001. *X*‐axis: time of survival in months; *Y*‐axis: estimated survival probability. Red line: ACU group; blue line: non‐ACU group. Numbers in red: number of patients at risk at that time in the ACU group; numbers in blue: number of patients at risk at that time in the non‐ACU group.

The ORs of levodopa therapy and nonmotor symptom medications were calculated between the ACU and non‐ACU groups using the aggregated medication records from onset to August 2018 or until the termination of the treatment. Kaplan–Meier curve analysis was performed to compare mortality over time between the two groups prior to matching, and the Cox proportional hazards model was used to estimate HRs for mortality with corresponding 95% CIs in which age groups, sex, and the duration of levodopa therapy were included in the analysis as covariates and for propensity score matching (PSM). The Kaplan–Meier analysis depicted in Figure 2 is based on the unmatched cohort and is reported for descriptive comparison; the primary inference for the survival outcome is based on the propensity score‐matched, covariate‐adjusted Cox proportional hazards model. Statistical significance was set at *p*‐value < 0.05. The proportional hazards assumption for the Cox model was evaluated using Schoenfeld residuals and log‐log survival plots for the primary exposure variable (ACU group) and each covariate; no material violations of the proportionality assumption were detected across the follow‐up period. All statistical analyses were performed using SAS version 9.3.

## 3. Results

### 3.1. Baseline Statistics

The final population of patients with PD who were initially diagnosed in the period from 2010 to 2011 was 46,908 patients after PSM analysis (Figure 1; ACU group = 23,454; non‐ACU group = 23,454). The study cohort was observed from the index date until August 2018. After the PSM analysis, no significant differences were found in the age groups, sex, economic status, or duration of levodopa therapy between the ACU and non‐ACU groups (Table 1). There were significant differences in the region of residence between populations. Our study cohort showed that > 35% of patients with PD lived in the Seoul or Gyeonggi Province, which is a suburban province around Seoul. The types of treatments received by patients in the ACU treatment group were acupuncture, electroacupuncture, moxibustion, cupping, and herbal formula (Supporting Table 2).

### 3.2. Survival

During the follow‐up period (range: 0–103 months; median: 87.9 months), the mortality rate was 26.01/1000 population/year in the ACU group and 28.91/1000 population/year in the non‐ACU group. The absolute difference was 2.90 per 1000 person‐years, corresponding to a cumulative absolute risk difference of approximately 2.1% points over the median follow‐up of 87.9 months, corresponding to an approximate number needed to be exposed of 48 over the study period, although this estimate should not be interpreted causally. The median survival was 90 months for the ACU group and 86 months for the non‐ACU group. In the Kaplan–Meier curve between two groups before matching, the patients in the ACU group had a slightly higher survival probability (log‐rank test, *χ* ^2^ = 1219.45, *p* < 0.001; Figure 2). Furthermore, crude and adjusted HRs for all‐cause mortality in patients with PD in Korea showed that patients who received acupuncture‐related therapies were associated with a slightly lower long‐term risk of all‐cause mortality with a crude HR of 0.96 (95% CI: 0.95–0.98) and adjusted HR of 0.90 (95% CI: 0.89–0.91) (Supporting Table 3). As the Kaplan–Meier curves shown in Figure 2 were generated from the unmatched cohort, they are presented for descriptive comparison only; the primary inference for the survival outcome relies on the propensity score‐matched, covariate‐adjusted Cox proportional hazards model reported above and detailed in Supporting Table 3.

### 3.3. Medication Use in ACU and Non‐ACU Groups

Comparisons of levodopa therapy between the ACU and non‐ACU groups are presented in Table 2. Medications used as levodopa therapy were levodopa (levodopa/carbidopa, levodopa/benserazide), levodopa + COMT inhibitors (levodopa/carbidopa/entacapone), and dopamine antagonists (bromocriptine, ropinirole, pramipexole). The ACU group showed reduced ORs of levodopa therapy (0.90, CI: 0.87–0.92). Particularly, the ORs of levodopa/carbidopa, levodopa/benserazide, levodopa/carbidopa/entacapone, ropinirole, and pramipexole were reduced in the ACU group. The OR of bromocriptine, however, showed no statistically significant difference.

**TABLE 2:** Odds ratios of receiving levodopa therapy during the follow‐up period (2010–2018) in the ACU group compared with the non‐ACU group (reference).

Class	Generic name	Non‐ACU group	ACU group	Non‐ACU group	ACU group	OR
		2010‐2011		2018 (∼before death)		
Levodopa	Levodopa/carbidopa	2584	2762	2416	2162	0.84 [0.76–0.92]^∗^
	Levodopa/benserazide	3857	3132	3181	2915	1.13 [1.06–1.20]^∗^

Levodopa + COMT inhibitors	Levodopa/carbidopa/entacapone	725	821	959	858	0.79 [0.65–0.93]^∗^

Dopamine antagonists	Bromocriptine	237	189	132	95	0.90 [0.58–1.23]
	Ropinirole	9567	8823	6621	5811	0.95 [0.91–1.00]^∗^
	Pramipexole	6883	7823	6565	5744	0.77 [0.72–0.82]^∗^

Total		23,853	23,550	19,874	17,585	0.90 [0.87–0.92]^∗^

*Note:* ACU: acupuncture‐related therapies; DTH: death of the patient. The OR represents the odds of receiving each medication during the entire follow‐up period (2010–2018, or until death or treatment termination) in the ACU group compared with the non‐ACU group (reference); an OR < 1 indicates lower odds of receiving that medication in the ACU group than in the non‐ACU group, whereas an OR > 1 indicates higher odds. The “2010‐2011” column reports the number of patients with at least one prescription of the listed medication during the index period (2010‐2011), and the “2018 (∼before death)” column reports the number of patients with at least one prescription of the listed medication during the final follow‐up year (2018) or in the year preceding death; these counts are descriptive and are not the inputs from which the OR was directly computed.

Abbreviation: OR, odds ratio.

^∗^ *p*‐value < 0.05.

Comparisons of medication use for nonmotor symptoms between ACU and non‐ACU groups are presented in Table 3. The ORs of the medications for insomnia, anxiety, and depression generally showed an increase (OR: 1.37, CI: 1.34–1.40), except for modafinil and desvenlafaxine, both of which had very low prescription frequencies. The overall ORs of medication use for sleeping disorders, anxiety, and depression were 1.45 [1.39–1.50], 1.58 [1.50–1.66], and 1.30 [1.22–1.39], respectively.

**TABLE 3:** Odds ratios of receiving medications for nonmotor symptoms during the follow‐up period (2010–2018) in the ACU group compared with the non‐ACU group (reference).

Target disease	Generic name	Non‐ACU group	ACU group	Non‐ACU group	ACU group	OR
		2010‐2011		2018 (∼before death)		
Sleeping disorders	Clonazepam	2474	1808	4531	4832	1.46 [1.39–1.53]^∗^
	Methylphenidate	157	106	165	163	1.46 [1.14–1.79]^∗^
	Zolpidem	776	739	6157	7013	1.20 [1.09–1.30]^∗^
	Modafinil	94	73	15	12	0.75 [0.00–1.57]
	Subtotal	3501	2726	10,868	12,020	1.45 [1.39–1.50]^∗^

Anxiety	Alprazolam	1341	1428	9633	11,643	1.58 [1.50–1.66]^∗^

Depression	Fluoxetine	412	315	378	423	2.58 [2.38–2.78]^∗^
	Nortriptyline	445	193	2328	2849	1.32 [1.01–1.63]^∗^
	Paroxetine	97	90	469	575	1.32 [1.01–1.63]^∗^
	Sertraline	167	73	323	359	2.54 [2.23–2.86]^∗^
	Venlafaxine	78	170	226	245	0.50 [0.17–0.82]
	Escitalopram	119	344	2186	2462	0.39 [0.17–0.61]
	Desvenlafaxine	0	0	19	23	—
	Subtotal	1318	1185	5929	6936	1.30 [1.22–1.39]^∗^

Total		9661	8065	37,298	42,619	1.37 [1.34–1.40]^∗^

*Note:* ACU: acupuncture‐related therapies; DTH: death of the patient. The OR represents the odds of receiving each nonmotor symptom medication during the entire follow‐up period (2010–2018, or until death or treatment termination) in the ACU group compared with the non‐ACU group (reference); an OR > 1 indicates higher odds of receiving that medication in the ACU group than in the non‐ACU group, whereas an OR < 1 indicates lower odds. The “2010‐2011” column reports the number of patients with at least one prescription of the listed medication during the index period (2010‐2011), and the “2018 (∼before death)” column reports the number of patients with at least one prescription of the listed medication during the final follow‐up year (2018) or in the year preceding death; these counts are descriptive and are not the inputs from which the OR was directly computed.

Abbreviation: OR, odds ratio.

^∗^ *p*‐value < 0.05.

## 4. Discussion

This study investigated the all‐cause survival and use of acupuncture‐related therapies in patients with PD diagnosed in 2010–2011 in Korea by following their medical records found in NHIS up to the time of patient’s death or August 2018. The results showed that the ACU group, or patients with records of acupuncture‐related therapies after the diagnosis of PD, showed a modest reduction in long‐term all‐cause mortality risk and received reduced rates of levodopa therapy. In contrast, the ACU group received more medications for nonmotor symptoms, including insomnia, anxiety, and depression, a trend found in nonmotor treatments, except for less frequently prescribed medications. To our knowledge, this is the first retrospective study with the largest PD cohort on a national level investigating the association between acupuncture‐related therapy use and both survival and medication treatments.

The results of the present study may reflect differences in prognosis or treatment patterns among patients with PD who used acupuncture‐related therapies. The ACU group showed reduced levodopa use compared with the non‐ACU group, which could potentially be explained by slower deterioration of motor symptoms and preservation of activities of daily living (ADL). Reduced levodopa use combined with a mild increase in survival probability may also reflect indirect effects of acupuncture‐related therapies through the prevention of complications such as aspiration pneumonia, falls, and fractures, which are major causes of morbidity and mortality in PD. This interpretation is partly supported by previous studies reporting lower prevalence of hospitalization due to pneumonia or sepsis [38] and reduced fracture risk [39] among acupuncture users with PD.

However, several alternative explanations for the lower levodopa use in the ACU group should be considered. Patients who seek acupuncture‐related therapies may have different treatment preferences, may delay pharmacologic escalation, or may have lower baseline disease severity. Therefore, the interpretation that reduced levodopa use reflects delayed disease progression remains speculative and cannot be directly inferred from the current data.

The ACU group showed significantly higher ORs of nonmotor symptom medication use for sleep disorders, depression, and anxiety. Psychiatric problems, such as depression, anxiety, and sleep disorders, worsen the quality of life and ADL in patients with PD [40–43]. An interpretation of this result is that patients with severe nonmotor symptoms exhibited a higher tendency to seek additional treatments, such as acupuncture, due to discomfort in daily living and lower quality of life, reflecting the patients’ unmet needs by prescribed medications. Therefore, we assume that in the PD population, patients who experienced more severe nonmotor symptoms were likely to seek acupuncture‐related therapies. Further investigation is required by matching patients between groups with similar levels of nonmotor symptom severity or medication dose.

Another possible interpretation is the inappropriate use of medications in patients with PD to manage nonmotor symptoms, an issue discussed by clinicians and researchers with an increasing rate in recent years. Previous studies indicated the initiation of medication use for nonmotor symptoms at an early stage of PD and long duration of use [26], patterns of multiple medication use [28], and possibly inappropriate use of medication [29, 30]. While the need is inevitable to manage nonmotor symptoms, overuse of such medications has been linked to additional problems, such as increases in the duration of hospitalization [28]. Taken together, it seems that patients with more severe nonmotor symptoms have a higher tendency to seek acupuncture‐related therapies, either to relieve nonmotor symptoms or to manage additional needs caused by medications for nonmotor symptoms. This pattern is consistent with confounding by indication, whereby patients with more severe nonmotor symptoms are more likely both to seek additional treatment including acupuncture‐related therapies and to require more nonmotor symptom medications. This alternative explanation cannot be excluded in the current study design and should be considered when interpreting the observed higher medication use in the ACU group.

The mechanism of acupuncture‐related therapies in PD has been discussed in a number of previous studies, many of which imply the synergistic impact of various treatment modalities, such as acupuncture, electroacupuncture, moxibustion, and herbal medicine. Acupuncture was the most commonly used treatment modality in many studies, including this study; both acupuncture and electroacupuncture were reported to improve motor symptoms, particularly gait disturbances, in patients with PD [44–47]. A previous study using functional magnetic resonance imaging showed that the mechanism of acupuncture in relieving PD symptoms might be modulation of the basal ganglia–thalamocortical circuit [17], in addition to systematic reviews and meta‐analyses that reported the effectiveness of acupuncture in both motor and nonmotor symptoms [48–53]. Moreover, acupuncture was reported to lower the risk of PD in patients with depression [54] and inflammatory bowel disease [55]. These studies suggest that acupuncture with its mechanism at the neural level not only relieves PD symptoms but also alleviates the factors involved in the mechanism of PD occurrence. Furthermore, an overview of reviews on the effects of herbal medicine on PD, whose quality of evidence was moderate to high, showed significant improvement in motor and nonmotor symptoms of PD, which were evaluated using the unified Parkinson’s disease rating scale (UPDRS) score, Webster scale score, Parkinson’s disease questionnaire (PDQ‐39), nonmotor symptoms questionnaire (NMSQuest), Chinese herbal medicine (CHM) syndrome integral scale, and Parkinson’s disease sleep scale (PDSS) [13]. Another review on moxibustion suggested that it could be helpful for relieving the symptoms of PD, although the evidence was not sufficient to draw a definitive conclusion [56].

From a neural mechanistic perspective, acupuncture‐related therapies may influence nonmotor symptoms in Parkinson’s disease (PD) through several pathways. Dysfunction of the basal ganglia–thalamocortical circuit in PD is implicated not only in motor control but also in mood regulation, cognitive processing, and sleep–wake cycling. Neuroimaging studies suggest that acupuncture can modulate activity within this circuit, providing a potential substrate for effects on nonmotor symptom domains [57]. In experimental models of PD, electroacupuncture has been reported to exert neuroprotective effects on dopaminergic neurons and to reduce neuroinflammatory markers, with associated improvements in cognitive function [58, 59]. Acupuncture has also been reported to influence serotonergic and noradrenergic neurotransmitter pathways relevant to anxiety and depression [60]. These mechanistic insights derive from prior experimental and clinical literature and cannot be inferred directly from the administrative data used in the present observational study, which did not include neurobiological endpoints. Rather, they provide a biologically plausible context for interpreting the observed patterns of medication use, suggesting that some patients may seek acupuncture‐related therapies to address nonmotor symptoms that remain insufficiently controlled by conventional treatment alone.

To our knowledge, this is the first study to investigate the association of acupuncture‐related therapy use with medication regimen and survival of Korean patients with PD at a national level. Prior NHIS‐based studies have examined either mortality outcomes in PD [10] or the effect of Korean medicine on PD incidence in specific comorbid populations, but none has jointly examined both the detailed medication regimen (including nonmotor symptom medications) and all‐cause survival in a propensity score‐matched national PD cohort. International cohort studies have similarly examined mortality or medication use in isolation. Considerable efforts were made to minimize the errors in subject selection by adding health insurance coverage expansion codes (V124) and prescription records of levodopa therapy. In previous studies using the NHIS cohort [38], PD was diagnosed based only on the disease classification record in the database. The addition of health insurance coverage expansion codes (V124) to identify eligibility of patients allowed for more accurate selection, since these codes, which secure medical expense assistance from NHIS, are provided with the consent based on the doctors’ medical decisions. In addition, records of levodopa therapy for > 7 days included a wide range of antiparkinsonian medications, such as levodopa, COMT inhibitors, dopamine antagonists, MAO‐B inhibitors, anticholinergics, and NMDA receptor antagonists. Finally, the duration of levodopa therapy was matched between groups to ensure that the two groups shared similar levels of symptom severity.

This study is also the first to examine the prescription records of levodopa therapy and nonmotor symptom medications in patients with PD at a national level. Although clinical symptoms of PD can be accurately evaluated using evaluation tools, such as UPDRS and NMSQuest, it is difficult to obtain such specific clinical information in retrospective studies using claims records, including the present study. Therefore, previous retrospective studies evaluated the effect of acupuncture‐related therapies on the overall prognosis of patients with PD, such as the hospitalization rate, mortality, and risk of fractures [38, 39]. While the importance of such prognoses cannot be overlooked, the disadvantage of these outcome measures remains substantial because major events such as death or hospitalization are not the only concerns in chronic neurodegenerative diseases, such as PD. Therefore, to overcome this limitation, this study followed the medication records and analyzed both levodopa therapy and nonmotor medications.

Limitations should be acknowledged. Most importantly, the NHIS data lack granular clinical measures such as detailed PD severity scores, disease subtypes (e.g., tremor‐dominant vs. postural instability/gait disorder), and comprehensive treatment histories. Second, the effects of individual components of herbs or acupuncture points could not be assessed in this study because such detailed treatment information is not consistently recorded in the NHIS database. In particular, herbal medicine exposure is likely substantially underestimated. A large proportion of herbal medicine prescriptions in Korea are dispensed outside the NHIS reimbursement system and therefore are not captured in claims data. In the present study, only 20% of patients in the ACU group were recorded as receiving herbal medicine; however, previous survey‐based research reported that more than 70% of Korean patients with PD use herbal medicine [21]. This discrepancy suggests potential exposure misclassification in the NHIS database, whereby patients who used herbal medicine may not have been recorded as such in the claims data. Because this misclassification is likely nondifferential with respect to outcomes, it may have attenuated the observed associations for the ACU group, meaning that the true exposure to herbal medicine could be substantially higher than that captured in this study. Finally, many patients received combinations of treatment modalities instead of a single modality, which only allowed the estimation of the general effect of acupuncture‐related therapies, and it was not possible to disentangle or statistically model the independent contribution of, or interaction between, individual components such as acupuncture, electroacupuncture, moxibustion, cupping, and herbal medicine. Factorial randomized controlled trials are needed to address this question.

Several additional limitations deserve explicit acknowledgement. First, the absence of standardized clinical severity measures (e.g., UPDRS scores, PD subtype classification, and functional status) in the NHIS administrative data means that PSM on age, sex, and levodopa duration could not fully account for differences in baseline disease burden between the ACU and non‐ACU groups. Residual confounding from these unmeasured variables is likely and may bias the observed associations in either direction. Second, the Cox proportional hazards assumption was verified using Schoenfeld residuals and log‐log survival plots, and no material violations were detected across the study period. Third, the observed reduction in levodopa use in the ACU group may reflect patient‐driven preferences, physician practice patterns in integrative medicine contexts, or lower baseline disease severity rather than a therapeutic benefit from acupuncture‐related therapies, and these alternative explanations cannot be excluded. Fourth, prescribing patterns and acupuncture utilization rates likely evolved across the study period (2010–2018) in parallel with changes in PD pharmacotherapy guidelines and Korean medicine reimbursement policy; these temporal trends represent a potential time‐varying confounder that future studies should address through period stratification. Fifth, the findings of this study may not be generalizable beyond Korea’s unique dual‐medical licensing system, where Korean medicine practitioners are state‐licensed and acupuncture‐related therapies are reimbursed under the NHIS; in most other healthcare systems, traditional medicine is less integrated and less accessible. Sixth, given the retrospective observational design of this study, the associations between acupuncture‐related therapy use and reduced mortality or levodopa use cannot be interpreted as evidence of a causal therapeutic effect. Furthermore, nonspecific (placebo and expectancy) effects of acupuncture‐related therapies cannot be separated from specific physiological effects; prospective randomized controlled trials incorporating sham‐acupuncture control arms are needed to isolate treatment‐specific effects. Seventh, future studies should incorporate subgroup analyses by age, sex, PD subtype, disease duration, and timing of acupuncture initiation to identify patient populations most likely to benefit.

In conclusion, this study showed that patients with records of using acupuncture‐related therapies after the diagnosis of PD were associated with a slightly lower long‐term risk of all‐cause mortality and with reduced rates of levodopa therapy. These patients received more medications for nonmotor symptoms, including insomnia, anxiety, and depression, a pattern that more likely reflects care‐seeking behaviour among patients with severe nonmotor symptoms or unmet medication needs than evidence of symptom management by acupuncture‐related therapies themselves. This study describes the observed prognosis of patients with PD depending on the utilization of acupuncture‐related treatments. These findings are associative in nature and should not be interpreted as evidence of direct therapeutic efficacy, as the observational design precludes causal inference and residual confounding is likely. Further studies are required to investigate the role of acupuncture in the management of nonmotor symptoms and medications for nonmotor symptoms. Prospective, randomized, sham‐controlled clinical trials with standardized clinical severity measures, patient‐reported nonmotor symptom outcomes, and adequate follow‐up are required to determine whether acupuncture‐related therapies confer specific clinical benefit beyond placebo in patients with PD.

## Author Contributions

Research project: conception: Bo‐Hyoung Jang, Seungwon Kwon, and Ye‐Seul Lee; organization: Bo‐Hyoung Jang, Seungwon Kwon, and Han‐Gyul Lee; execution: Ye‐Seul Lee.

Statistical analysis: design: Bo‐Hyoung Jang and Ye‐Seul Lee; execution: Ye‐Seul Lee, Han‐Gyul Lee, and Hi‐Joon Park; review and critique: Bo‐Hyoung Jang, Seungwon Kwon.

Manuscript preparation: writing of the first draft: Ye‐Seul Lee; review and critique: Bo‐Hyoung Jang, Seungwon Kwon, Hi‐Joon Park, and Han‐Gyul Lee.

## Funding

This research was supported by the National Research Foundation of Korea (NRF) grant funded by the Korea Government (MSIT; Nos. 2018R1D1A1A02086175 and NRF‐2022M3A9B6017813).

## Conflicts of Interest

The authors declare no conflicts of interest.

## Supporting Information

Additional supporting information can be found online in the Supporting Information section.

## Supporting information


**Supporting Information** Supporting information is provided as a separate file. Supporting Table 1 summarizes the antiparkinsonian medications used to define the study cohort. Supporting Table 2 presents the types of acupuncture‐related therapies received by patients in the ACU group. Supporting Table 3 reports the crude and adjusted HRs for all‐cause mortality in the propensity score‐matched cohort.

## Data Availability

The data that support the findings of this study are available on request from the corresponding author. The data are not publicly available due to privacy or ethical restrictions.
