# Supplementary material for: Acupuncture‐Related Therapies as a Potential Adjuvant Option for Parkinson’s Disease: Effects on Symptom Management, Medication Use, and Mortality
Source: Parkinsons Dis. 2026 Jun 17;2026:5512318. doi: 10.1155/padi/5512318 (PMC13273529; doi:10.1155/padi/5512318)
Supplement: Supplementary file 1 — Supporting Information Supporting information is provided as a separate file. Supporting Table 1 summarizes the antiparkinsonian medications used to define the study cohort. Supporting Table 2 presents the types of acupuncture‐related therapies received by patients in the ACU group. Supporting Table 3 reports the crude and adjusted HRs for all‐cause mortality in the propensity score‐matched cohort. [file PADI-2026-5512318-s001.doc]

**Supporting Table 1. Types of levodopa therapy medications examined to define the study cohort.**

| **Class** | **Generic Name** |
| --- | --- |
| Levodopa | Levodopa/Carbidopa |
|  | Levodopa/Benserazide |
| Levodopa + COMT inhibitors | Levodopa/Carbidopa/Entacapone |
| Dopamine antagonists | Bromocriptine |
|  | Ropinirole |
|  | Pramipexole |
|  | Rotigotine |
| MAO-B inhibitors | Selegiline |
|  | Rasagiline |
| Anticholinergics | Trihexyphenidyl |
|  | Benztropine |
|  | Procyclindine |
|  | Biperiden |
| NMDA receptor antagonists | Amantadine |

**Supporting Table 2. Types of treatments received by ACU-treated patients in Parkinson's disease cohort.**

|  | **Number of patients** | **%** |
| --- | --- | --- |
| **Acupuncture** | 23,454 | 100% |
| **Electroacupuncture** | 23,454 | 100% |
| **Moxibustion** | 10,001 | 43% |
| **Cupping** | 6,677 | 28% |
| **Herbal formula** | 4,753 | 20% |

**Supporting Table 3.** Crude and adjusted hazard ratios of all-cause mortality in propensity score-matched Parkinson’s disease cohort with and without ACU treatments

|  | **Crude HR [95% CI]** | ***P*-value** | **Adjusted HR [95% CI]** | ***P*-value** |
| --- | --- | --- | --- | --- |
| **Mortality** | 0.96 [0.95 - 0.98] | < 0.001 | 0.90 [0.89 - 0.91] | < 0.001 |
